# Response letter to “Confounding by indication and exposure misclassification may undermine corticosteroid effect estimates in ICU patients with alcohol-related hepatitis”

**DOI:** 10.1186/s13613-025-01487-3

**Published:** 2025-05-24

**Authors:** Guillaume Dumas, Maxime Gasperment, Hafid Ait-Oufella

**Affiliations:** 1 Université Grenoble Alpes, Grenoble, France; 2 Service de Médecine Intensive Réanimation - CHU Grenoble Alpes, Grenoble, France; 3 3- Service de Médecine Intensive Réanimation, Assistance Publique - Hôpitaux de Paris (AP-HP), Hôpital Saint-Antoine, 184 Rue du Faubourg Saint-Antoine, Paris Cedex 12, 75571 France; 4 Sorbonne Université, Paris, France

To the Editor:

We thank Drs Mao and Li [1]. for their interest in our study [2]. In their comment, Drs Mao and Li highlight certain methodological points that could have affected the estimation of the effect of corticosteroids on the prognosis of patients with severe alcohol related hepatitis. They correctly point out the difficulties of making causal inferences from real-world data, and we fully agree that our findings cannot be understood as a plea against using corticosteroids in this situation.

However, we would like to offer additional perspective to enhance the interpretation of our findings. The main objective of this work was to describe the clinical picture of patients admitted to the intensive care unit with a diagnosis of alcohol related hepatitis, for which little data is available, and not to evaluate the benefit of corticosteroids use in severe alcohol related hepatitis, for which it remains a recommended treatment, although the target population is still poorly defined [3, 4]. In this regard, our study emphasizes the wide diversity of clinical trajectories and the heterogeneity of reasons for ICU admission in these patients.

Regarding the analysis of corticosteroids effect, while we did not frame our study as an emulated target trial (which typically requires a large patient sample), we adhered to several guidelines to account for the biases inherent to observational and retrospective data [5]. First, we included only patients with a Maddrey score ≥ 32 and a MELD score ≥ 20, which are the thresholds commonly used in clinical trials. Second, to limit survivorship bias, only patients at risk who received steroids no more than 3 days prior to ICU admission were retained in the final analysis. Last, all potential confounders previously described in the literature (including patient severity and ongoing infection) were included in the propensity score. Although treatment assignment in ICU settings is rarely random and the assumption of strong ignorability may not hold, we believe we have minimized these pitfalls. To evaluate if this estimate is potentially biased by unmeasured confounding, we applied the E-value, which we estimated at 1.40. Therefore, an unmeasured confounder would need a hazard ratio of at least 1.40 with both corticosteroids use and survival to explain the observed hazard ratio of 1.13 [6]. Interestingly, in the subgroup of patients who underwent a liver biopsy, the estimated E-value for the hazard ratio was 4.87, suggesting a robust association. Finally, we found that corticosteroids responders according to Lille score had significantly better survival, which advocates for the use of this medication in these patients, but with an early evaluation of treatment response.

Thus, from our point of view, our data should not be interpreted as an advocacy against corticosteroids use but highlight two important findings, namely the likely difficulties in establishing a definitive diagnosis of alcohol related hepatitis based solely on clinical criteria in this population (which often presents with multiple intertwined causes of organ dysfunction), and the necessity to better identify those who could benefit the most from this treatment. Liver biopsy could provide valuable assistance in this challenging context.
